# Supplementary material for: Low prevalence of helminth infection in Ugandan children hospitalized with severe malaria
Source: PLoS One. 2025 Sep 11;20(9):e0332246. doi: 10.1371/journal.pone.0332246 (PMC12425322; doi:10.1371/journal.pone.0332246)
Supplement: S1 Text — (DOCX) [file pone.0332246.s001.docx]

S1 Text. Molecular Methods

We purchased custom TACs produced by ThermoFisher Scientific (Waltham, MA). TAC is a 384-well array card with 8 ports for loading samples and each 1.5-µL well contains dried-down primers and hydrolysis probes for the detection of defined targets. All assays contained the same concentrations of primers (900 nmol/L) and probe (250 nmol/L).

For analysis, we mixed 40 μL of template (0.6 μL total template per reaction well) with 60 μL of AgPath-ID™ One-Step RT-PCR Reagents (Quantabio, Beverly, MA), Combined positive controls were developed using methods from Kodani *et al.* 2012. Following the manufacturer’s instructions, we centrifuged each card twice at 1,200 rpm for one minute, sealed the card, trimmed the loading ports, and loaded the card into a QuantStudio 7 Flex Instrument (Thermo Fisher Scientific, Waltham, MA). We used the following thermocycling conditions: 95°C for 10 min, followed by 45 cycles of 95°C for 15s and 60°C for 1 min.

A plasmid with all targets was designed and ordered (Twist Bioscience), which we used for a standard curve and as a PCR positive control. Results of the standard curve were within the expected range (S1 Table). All PCR positive controls amplified as expected and we detected the positive extraction control (i.e., bovine herpes virus) in all samples. Using the Quantstudio Real-Time PCR software (Thermo Fisher Scientific, Waltham, MA) we plotted ΔRn (y-axis) vs. cycle number (x-axis) for each assay and set the threshold (on the y-axis) at the point where the positive control began exponential amplification. Any positive amplification after a Cq of 40 was called a negative to reduce the potential for false positives.
