# Supplementary material for: Low prevalence of helminth infection in Ugandan children hospitalized with severe malaria
Source: PLoS One. 2025 Sep 11;20(9):e0332246. doi: 10.1371/journal.pone.0332246 (PMC12425322; doi:10.1371/journal.pone.0332246)
Supplement: S1 Table — (DOCX) [file pone.0332246.s002.docx]

S1 Table. Assay performances

| Target | R^2^ | slope | efficiency | y-intercept | ref | LOD* (gc/µL) |
| --- | --- | --- | --- | --- | --- | --- |
| *Ancylostoma duodenale* | 0.999 | -3.2636 | 102% | 37.788 | [1] | 10^2^ |
| *Ascaris lumbricoides* | 0.999 | -3.1673 | 107% | 36.312 | [1] | 10^2^ |
| Bovine herpes virus (BHV) | 0.996 | -3.0444 | 113% | 35.118 | [2] | 10^2^ |
| *Necator americanus* | 0.997 | -3.2457 | 103% | 39.44 | [1] | 10^2^ |
| 16S rRNA | 0.998 | -3.1537 | 108% | 35.397 | [3] | 10^2^ |
| *Schistosoma mansoni* | 0.999 | -3.191 | 106% | 36.398 | [1] | 10^2^ |
| *Strongyloides stercolaris* | 0.998 | -3.109 | 110% | 36.52 | [1] | 10^2^ |
| *Trichuris trichiura* | 0.999 | -3.1886 | 106% | 36.88 | [1] | 10^2^ |

*This was the lowest concentration assessed on our standard curve, and yielded positive amplification for all targets

| Target | Gene | Assay |
| --- | --- | --- |
| *Ancylostoma duodenale* | *ITS2* | Fwd: GAATGACAGCAAACTCGTTGTTG  Rev: ATACTAGCCACTGCCGAAACGT  Probe: ATCGTTTACCGACTTTAG |
| *Ascaris lumbricoides* | *ITS1* | Fwd: GCCACATAGTAAATTGCACACAAAT  Rev: GCCTTTCTAACAAGCCCAACAT  Probe: TTGGCGGACAATTGCATGCGAT |
| Bovine herpesvirus | BHV | Fwd: GAGCAAAGCCCCGCCGAAGGA  Rev: TACGAACAGCAGCACGGGCGG  Probe: GAACCTGCCCACGCGCTGAAAC |
| *Necator americanus* | *ITS2* | Fwd: CTGTTTGTCGAACGGTACTTGC  Rev: ATAACAGCGTGCACATGTTGC  Probe: CTGTACTACGCATTGTATAC |
| 16S rRNA | 16S | Fwd: ATGGYTGTCGTCAGCT  Rev: ACGGGCGGTGTGTAC  Probe: CAACGAGCGCAACCC |
| *Schistosoma mansoni* | Dispersed repetitive sequence | Fwd: GGTCTAGATGACTTGATYGAGATGCT  Rev: TCCCGAGCGYGTATAATGTCATTA  Probe: TGGGTTGTGCTCGAGTCGTGGC |
| *Strongyloides stercolaris* | Dispersed repetitive sequence | Fwd: TCCAGAAAAGTCTTCACTCTCCAG  Rev: TGCGTTAGAATTTAGATATTATTGTTGCT  Probe: TCAGCTCCAGTTGAACAACAGCCTCCAA |
| *Trichuris trichiura* | 18S rRNA | Fwd: TTGAAACGACTTGCTCATCAACTT  Rev: CTGATTCTCCGTTAACCGTTGTC  Probe: CGATGGTACGCTACGTGCTTACCATGG |

References

1. Liu J, Gratz J, Amour C, Nshama R, Walongo T, Maro A, et al. Optimization of Quantitative PCR Methods for Enteropathogen Detection. Chan KH, editor. PLoS One. 2016;11: e0158199. doi:10.1371/journal.pone.0158199

2. Wang J, O’Keefe J, Orr D, Loth L, Banks M, Wakeley P, et al. Validation of a real-time PCR assay for the detection of bovine herpesvirus 1 in bovine semen. J Virol Methods. 2007;144: 103–108. doi:10.1016/j.jviromet.2007.04.002

3. Ritalahti KM, Amos BK, Sung Y, Wu Q, Koenigsberg SS, Löffler FE. Quantitative PCR Targeting 16S rRNA and Reductive Dehalogenase Genes Simultaneously Monitors Multiple *Dehalococcoides* Strains. Appl Environ Microbiol. 2006;72: 2765–2774. doi:10.1128/AEM.72.4.2765-2774.2006

4. Bangirana P, John CC, Idro R, Opoka RO, Byarugaba J, Jurek AM, et al. Socioeconomic Predictors of Cognition in Ugandan Children: Implications for Community Interventions. PLoS One. 2009;4: e7898. doi:10.1371/journal.pone.0007898
