# Supplementary material for: Low prevalence of helminth infection in Ugandan children hospitalized with severe malaria
Source: PLoS One. 2025 Sep 11;20(9):e0332246. doi: 10.1371/journal.pone.0332246 (PMC12425322; doi:10.1371/journal.pone.0332246)
Supplement: S2 Table — (DOCX) [file pone.0332246.s003.docx]

S2 Table. Regression analysis

| Independent Variable | Reference | Risk Ratio (95% CI) | Adjusted Risk Ratio (95% CI) |
| --- | --- | --- | --- |
| ≥1 helminth infection | No helminths detected | 1.03 (0.87, 1.23) | 1.04 (0.82, 1.31) |
| Age: 13-24 months | Age: ≤12 months | 0.98 (0.86, 1.10) | 0.97 (0.84, 1.12) |
| Age: 25-48 months |  | 0.93 (0.82, 1.05) | 0.93 (0.82, 1.05) |
| SES: Quantile 2 | SES: Quantile 1 | 1.01 (0.93, 1.14) | 1.02 (0.91, 1.14) |
| SES: Quantile 3 |  | 0.95 (0.81, 1.10) | 0.94 (0.80, 1.10) |
| SES: Quantile 4 |  | 0.99 (0.86, 1.13) | 0.98 (0.85, 1.12) |
| SES: Quantile 5 |  | 0.88 (0.73, 1.06) | 0.86 (0.69, 1.06) |

Note: SES was determined using a previously validated scoring metric [4]

Model Diagnostics

While traditional measures of model fit (e.g., AIC or likelihood-ratio tests) are not directly available for GEE models due to their quasi-likelihood framework, we conducted diagnostic checks using a comparable Poisson GLM with robust standard errors to evaluate residual patterns and multicollinearity.

Diagnostic plots of deviance and Pearson residuals showed no evidence of strong outliers, skewed distributions, or model misspecification. Residuals were generally centered around zero, with no patterns suggesting poor model fit. Additionally, we assessed multicollinearity using variance inflation factors (VIFs). All adjusted VIFs were close to 1, indicating that collinearity among predictors was not a concern. These findings suggest that the model assumptions were reasonable and that our results are not likely biased by model misfit or predictor redundancy.
