# Supplementary material for: Low prevalence of helminth infection in Ugandan children hospitalized with severe malaria
Source: PLoS One. 2025 Sep 11;20(9):e0332246. doi: 10.1371/journal.pone.0332246 (PMC12425322; doi:10.1371/journal.pone.0332246)
Supplement: Attachment — Submitted filename: report_10.1101+2024.10.07.24314986.pdf [file pone.0332246.s007.pdf]

ScreenIT Report

This is an automated report for 10.1101+2024.10.07.24314986.pdf. This report was solicited by the PLOS One editorial team and provided by ScreenIT.

ScreenIT is an independent group of scientists developing automated tools that analyze academic papers. A set of automated tools screened your submitted manuscript and provided the report below. Each tool was created by your academic colleagues with the goal of helping authors.

The tools look for factors that are important for transparency, rigor and reproducibility, and we hope that the report might help you to improve reporting in your manuscript. Within the report you will find links to more information about the items that the tools check. These links include helpful papers, websites, or videos that explain why the item is important.

While our screening tools aim to improve and maintain quality standards they may, on occasion, miss nuances specific to your study type or flag something incorrectly. Each tool has limitations that are described on the ScreenIT website. The tools screen the main file for the paper; they are not able to screen supplements stored in separate files.

Please note that the Academic Editor had access to these comments while making a decision on your manuscript. The Academic Editor may ask that issues flagged in this report be addressed.

If you would like to provide feedback on the ScreenIT tool, please email the team at ScreenIt@bih-charite.de. If you have questions or concerns about the review process, please contact the PLOS One office at plosone@plos.org.

How to read the report:

- no action required

? - review for potential issues

! - a critical issue was found by a tool

n/a - may not be applicable to your paper

- more information about this item can be obtained using this link

Rigor

n/a	Ethics	not required.
✓	Inclusion and Exclusion Criteria	*"Full details of study enrollment, inclusion and exclusion criteria and study procedures have been previously published [17]."*
?	Flow charts and attrition	We did not find a study flow chart of excluded observations. We strongly recommend using flow charts because they provide an overview of the study design and more information about attrition. If you included a study flow chart in your supplemental files, we apologize for missing this. Our tool is not able to screen separate supplemental files.<br><br>Sentence about attrition: *"not required."*
?	Sex as a biological variable	Not detected. Please consider reporting the sex of human participants, animals or cells, if you haven't done so.
✓	Subject Demographics	Age: *"Methods We prospectively enrolled 600 children with severe malaria and 120 community children 6 months to 48 months of age at Mulago National Referral Hospital in Kampala and Jinja Regional Referral Hospital in Jinja, Uganda."*
?	Randomization	Not detected. If you performed an experimental study, please specify whether participants, animals or samples were randomly assigned to treatments or groups, and specify the randomization procedure.
?	Blinding	Not detected. Please specify whether blinding was used at various phases of the experiment (e.g., blinding of patients, caregivers, outcome assessments, data analysis).
✓	Power Analysis	*"A sample size of 177 children with severe malaria and 25 community control children provided 80% power to detect a 17% absolute difference in intestinal helminth infection rates in the children with severe malaria, if the baseline rate of stool helminth infection in the control group was 20% [8,18]."*

n/a	Replication	not required.

Generated by Barzooka (flow charts), SciScore (everything else).
SciScore only screens the text of the methods section; we apologize if the tool has missed items reported elsewhere in the paper or supplements.

Resources

Persistent unique identifiers for research resources, RRIDs, can be used to track these key resources though the literature. They are requested or required in many journals, but even if your journal does not require them, we strongly encourage the use of these for the readers of papers who wish to easily track down the key materials like antibodies, software tools, or mice.

Software and Algorithms	*"We generated summary statistics using Microsoft Excel 365 and performed statistical analyses in RStudio (R Foundation for Statistical Computing, Vienna, Austria"*	? Microsoft Excel suggested: Microsoft Excel, RRID:SCR_016137. Please make sure this RRID suggestion is accurate before including in your manuscript.

Generated by SciScore.
SciScore only screens the text of the methods section; we apologize if the tool has missed items reported elsewhere in the paper or supplements.

Transparency

?	Open code	If you wrote code to analyze your data, please consider sharing the code in a public repository. This makes it easier for others to reproduce your analyses, and may also aid others seeking to analyze similar datasets.

?	Open data	If permitted, sharing data on a public repository with a persistent identifier (a DOI or accession number) can improve reproducibility and make it easier for other scientists to expand on your work. Papers with open data are cited more often than papers without open data. Some institutions have an expert who can provide advice on data sharing.
✓	Self-acknowledged limitations	*"Study limitations include a small sample size, which permitted detection of only large differences in stool helminth infection rates between children with severe malaria and community children. In addition, this studys sample size was much smaller than those of helminth surveillance studies in Uganda [24]. Study strengths include the rigorously characterized cohort of children with severe malaria and the use of modern molecular methods with a higher sensitivity than microscopy [34]. Future work on the relation between intestinal helminth infection and malaria in communities with a higher prevalence of helminth infection will be important to better elucidate the relationship between these pathogens. However, the study results suggest that in Kampala and Jinja, stool helminth infection is rare and therefore an unlikely contributor to risk of severe malaria."*
?	Conflict of interest statement	It is important to supply a conflict of interest statement, even if there are none to declare, to transparently present the research.
✓	Funding statement	Thank you for reporting your funding source(s), or stating that there was no specific funding for this work.
n/a	Registration statement	A registered protocol makes the planned research project more transparent and can protect against bias. Please consider registering future confirmatory studies.

Generated by ODDPub, limitation-recognizer, rtransparent, trial-identifier.

Figures

	No bar graphs of continuous data	Thank you for not using bar graphs to present continuous data. Many different datasets and data distributions can lead to the same bar graph, and the actual data may suggest different conclusions from the summary statistics alone.
	No rainbow color maps	Thank you for not using rainbow colormaps. Rainbow color maps distort readers' perception of the data by introducing visual artifacts and are not accessible to colorblind readers.

Generated by JetFighter, Barzooka.
