# Supplementary material for: Low prevalence of helminth infection in Ugandan children hospitalized with severe malaria
Source: PLoS One. 2025 Sep 11;20(9):e0332246. doi: 10.1371/journal.pone.0332246 (PMC12425322; doi:10.1371/journal.pone.0332246)
Supplement: Attachment — Submitted filename: Response to PLOS NTDs.docx [file pone.0332246.s006.docx]

Response to PLOS NTDs Review

Reviewer #1

| Reviewer Comment | Action Taken | Response |
| --- | --- | --- |
| The problem, objectives, methods and discussion don't seem to align. The aim is to find the prevalence but the methods don't mention how this will be done | We added headers to the methods section (see line 98 and 120) | We describe the methods for analyzing stool samples for helminth DNA starting at line 120. |
| The study design was not clearly defined and the ratio of 5 cases to 1 control was not explained and how the bias introduced with such a ratio would be addressed. | We added explanatory text (see line 100-101). | We direct the reader to reference 17, which has a more detailed description of the study and enrollment. We recognize additional detail is useful for clarity and have provided additional detail. |
| ) There is a mention of prospective enrollment, but this is not explained nor is there explanation on the sampling method for both groups. | We have added reference 18 to lines 103 and 105. | We briefly describe enrollment in lines 99-114. Additional information is described in references 17 and 18. |
| There is no mention of criteria used to subset the sample from the children from the community | We added that the children were age matched to children with severe malaria (line 109) | The inclusion and exclusion criteria are listed on lines 108-114. |
| Poor method section makes analysis of the results difficult |  | We politely disagree. We adhered to MIQE guidelines for reporting qPCR data (see S1 Text, S1 Table). |
| There seems to be a disconnect between the aim and conclusion |  | We politely disagree. Our aims were to assess helminth prevalence and to assess helminths modified the risk of severe malaria. The first sentence of the discussion, Line 178, is clearly supported by our results. |
