# Supplementary material for: Low prevalence of helminth infection in Ugandan children hospitalized with severe malaria
Source: PLoS One. 2025 Sep 11;20(9):e0332246. doi: 10.1371/journal.pone.0332246 (PMC12425322; doi:10.1371/journal.pone.0332246)
Supplement: Attachment — Submitted filename: Response to Reviewers_v2.docx [file pone.0332246.s009.docx]

Reviewer 2

We thank Reviewer #2 for their detailed and thoughtful feedback on the manuscript. Reported line numbers correspond to the clean version of the manuscript.

| Comment | Action Taken | Response |
| --- | --- | --- |
| **Abstract** |  |  |
| The title refers only to soil-transmitted helminths, but the study also includes *Schistosoma mansoni*, which is not classified as an STH. Consider revising the title to more accurately reflect the full range of intestinal helminths tested. | Revied the title  Line 1  Low prevalence of helminth infection in Ugandan children hospitalized with severe malaria | Agreed |
| It is unclear whether the community control children were tested for *Plasmodium* spp. infections. Were these controls truly malaria-negative, or simply asymptomatic? | Revised  Line 23  We analyzed 177 stool samples from a cohort of children with severe malaria enrolled in two hospitals in Uganda from 2014-2017, all 6 months to 48 months of age. We also analyzed 25 stool samples from community children who were negative for malaria via rapid diagnostic test and were enrolled from the same household or neighborhood and matched by age, sex, and time of enrollment. | These 25 community control children were negative for malaria, which was determined using a rapid diagnostic test. |
| The use of children from the “same household or neighborhood” is reasonable, but this detail should be explained more thoroughly to ensure that the reader understands how confounding factors were minimized. | See previous response | Agreed. We now state that children were matched by age, gender, and time of enrollment. |
| The small number of controls (n=25 vs. 177 cases) also raises concerns about statistical power and should be mentioned or justified. |  | We report our sample size calculation on line 145. We have not included this justification in the abstract to keep the abstract concise. |
| The methods for helminth detection are well-described (stool sample testing using qPCR), but more clarity is needed on the malaria diagnosis—was it based on clinical presentation, lab confirmation, or both? | Revised  Line 24  All children were 6 months to 48 months of age and had a clinical presentation of and laboratory confirmation for severe malaria. | Agreed. It was both. |
| The adjusted risk ratio (aRR = 1.0) and wide confidence interval suggest no effect, but the meaning of these statistics should be clearer for a broad audience. A plain-language interpretation would help, e.g., “Helminth infection did not increase or decrease the likelihood of severe malaria.” | Revised  Helminth infection did not increase or decrease the risk of severe malaria in this cohort (aRR = 1.0, 95% Confidence Interval = 0.82, 1.3, p = 0.78). | Agreed |
| The concluding sentences introduce a key context (the deworming program in Uganda) which seems important but is not tied explicitly to the study’s findings until the end. Consider stating this earlier to help frame the findings. | We moved this sentence to earlier in the abstract. | Agreed |
| Also, "not a major contributor to risk" is vague—based on the data, it's more accurate to say helminths were not associated with severe malaria in this sample. | Revised  Line 35  In these areas of Uganda, the national deworming campaign has been highly successful, as stool-based helminth infection was rare even when using sensitive methods of detection and helminths were not associated with severe malaria in this study. |  |
| **Introduction** |  |  |
| The rationale for the study—that helminths might modulate malaria risk—is acknowledged but underexplored. A few sentences explaining potential biological mechanisms (e.g., immune modulation by chronic helminth infection) would strengthen the logical bridge to the study aims. | Revised  Line 90  Helminth infection often modulates host immunity by shifting immune response towards a Th2-dominant profile and inducing regulatory pathways that suppresses inflammation [17,18]. This presents two contrasting possibilities. Immune modulation may limit an effective Th1-mediated response necessary to control infection by P. falciparum, while helminth-driven immunoregulation may mitigate immunopathology associated with severe malaria. Empirical data on helminth and *P. falciparum* infection may provide further insight into these interactions. | This is a very helpful addition to the introduction. |
| The line “determine if intestinal helminths modified risk of severe malaria” is somewhat vague. Clarify how modification of risk is measured—i.e., by comparing prevalence of helminths between cases and controls, and using adjusted risk ratios. | Revised  Line 99  determine if intestinal helminths modified risk of severe malaria in hospitalized pediatric patients compared to community controls enrolled in this study. | Revised |
| The introduction of the “Neurodevelopment Outcomes in Children with Severe Malaria Study” (NDI) lacks context. Readers unfamiliar with the study may be confused by its sudden mention and unclear relevance to the helminth-malaria research question. A brief summary of what the NDI study is, and why it provides a suitable population for this analysis, would be helpful. | Revised  Line 107  The primary outcome of the NDI study was to assess cognitive outcomes in children 12-months following hospitalization with severe malaria compared to matched controls. | Agreed |
|  |  |  |
| Methods |  |  |
| The description of cohort enrollment is convoluted and difficult to follow. Please clarify whether 600 children with severe malaria were enrolled or whether 600 children total were enrolled across both groups. The breakdown by group and recruitment site should be clearly tabulated. | We revised this paragraph for clarity.  Line 108  We prospectively enrolled 600 children with severe malaria and 120 community children 6 months to 48 months of age at Mulago National Referral Hospital in Kampala and Jinja Regional Referral Hospital in Jinja, Uganda from 28/03/2014 to 17/04/2017 [19,20]. | This was a sub-study of the overall study and did not include the entire cohort of children. We include references 17 and 18 where full details of study enrollment may be found. |
| It is not clear whether community controls were tested for *Plasmodium* spp., or if they were assumed uninfected based on lack of symptoms. This distinction is crucial for interpreting any analysis of “risk modification.” | Revised  Line 119  Community control children were tested for asymptomatic *Plasmodium* infection via a rapid diagnostic test (RDT).  Line 137  and (3) community control children were negative for *Plasmodium* infection via RDT. | This is a very important comment. |
| The phrase “nuclear family, extended family, or household area” is imprecise. Clarify whether community controls were biologically related, shared the household, or were simply neighborhood-matched. | Revised  Line 119  Community children were enrolled from biological siblings or were neighborhood-matched to enrolled children. with severe malaria. Controls were enrolled to be within the same age range as the children with severe malaria. | We appreciate the need for clarity in terminology |
| The selection of the 202 stool samples appears arbitrary, as the manuscript does not report how many total samples were available or how many were excluded based on timing or quantity. It is also unclear whether the final sample is representative of the broader cohort or if exclusions may have introduced bias. | Revised  Line 134  We randomly selected a subset of 202 stools from the 502 samples available for inclusion in this study based on the following criteria: (1) the stool sample was collected ≤48 hours after admission (only for children with severe malaria); (2) adequate stool was available for nucleic acid extraction (i.e., ≥100mg); and (3) community control children were negative for *Plasmodium* infection via RDT. These criteria included fecal samples from 177 children with severe malaria and 25 community controls.  Line 140  Among the 177 children with severe malaria, 16 presented with cerebral malaria, 39 with respiratory distress, 60 with multiple seizures, 32 with severe malaria anemia, and 30 with prostration. Among all severe malaria children in the NDI study, 7.3% died. However, mortality was less frequent among the children (2.3%, n=4/177) included in this study because we were not able to obtain fecal samples from many of the children who died.  Line 264  Mortality prevalence was lower among children with severe malaria in this study compared to the parent NDI study. Our analysis may have missed some of the high severity cases. |  |
| The molecular methods appear rigorous, but the repeated use of “highly sensitive” should be supported by data or comparison to alternative detection methods. What was the lower limit of detection for each helminth species? | We have included the LOD in Table S1  Line 235  qPCR has repeatedly been shown to be more sensitive than microscopy for diagnosing helminth infections, particularly in cases of low-intensity infections [36–38]. | We appreciate the need to report the LOD. |
| A table of primer targets or detection thresholds would enhance transparency (either in manuscript or supplementary materials). | We have included this information in Table S1. |  |
| The power calculation is based on a 20% helminth prevalence drawn from outdated data (1998-2005) on school-aged children, which does not reflect the current study population of 6–48-month-olds or account for the impact of Uganda’s national deworming program (started in 2003 expanded in 2005 through Child Health Days). Your own findings show a much lower prevalence, suggesting the study may be underpowered to detect the expected effect size. This weakens the basis for interpreting a null association. A post hoc power calculation using observed prevalence or a sensitivity analysis would help clarify the strength of your conclusions. | Revised  Line 263  Our study was underpowered to differentiate between small differences in prevalences, as we observed here. | This is an important limitation that we now acknowledge in the Discussion section. |
| The use of Poisson regression should be justified. If it was used to estimate relative risks for a binary outcome, did the authors apply a version of the model that adjusts for overdispersion or variance mis-specification? Given the binary nature of the outcome (severe malaria: yes/no), logistic regression may have been more appropriate and more conventional. | Revised and included references 26 and 27.  Line 171  We used generalized estimating equations (*geepack* package) to fit Poisson regression models with robust standard errors and an exchangeable correlation structure [26]. We assessed if infection by ≥1 helminth modified risk of severe malaria [27]. | We now provide further detail on the model we used. When defensible, we prefer to report risk ratios instead of odds ratios because odds ratios are frequently misinterpreted as risk ratios in the health sciences (Ref 27). Although Poisson models were originally designed for count data, the use of robust standard errors corrects for the variance mis-specification that arises from applying this model to binary outcomes. |
| Socioeconomic status is included as a covariate but not defined. How was it measured or categorized? | We clarified these covariates  Line 173  helminth infection (binary), age (categorical: <12 month, 13-24 months, >24 months), and socioeconomic status (quantile) were included as independent variables. |  |
| The use of the term "ova concentration" in Table 1 is misleading given that the study employed PCR-based methods, which detect parasite DNA, not eggs. PCR quantifies nucleic acid regardless of whether it originates from eggs, larvae, or adult worms, and does not provide visual confirmation of ova as microscopy does. It would be more appropriate and scientifically accurate to report these results as "parasite DNA concentration" or "gene copies per gram of feces." This correction is important to avoid confusion about what was actually measured and how the findings should be interpreted. | Revised | Agreed |
| The use of t-tests to compare helminth prevalence and gene copy concentrations between sites (e.g., Kampala vs. Jinja) is not described in the methods and appears inconsistent with the stated statistical approach. Given that the authors accounted for clustering by site in their Poisson regression model, it is unclear why additional unadjusted site-level comparisons were performed. | We deleted this column | Agreed. |
| The Poisson regression model results are briefly presented, and the main finding—a non-significant association (aRR = 1.0, p = 0.78)—is clearly stated. However, the model output is summarized without context. For example, the confidence interval includes both potential protective and harmful associations, indicating high uncertainty. There is no discussion of model fit, multicollinearity, or residuals. In the supplementary table, multiple socioeconomic strata are included, but no explanation is given for how these were measured or categorized. Without that detail, it’s difficult to assess whether the covariate adjustment is meaningful or adequately controlled for confounding. | We revised the methods to provide additional detail and include reference 28  Line 173  In our model, severe malaria status was the dependent variable, while helminth infection (binary), age (categorical: <12 month, 13-24 months, >24 months), and socioeconomic status (quantile) were included as independent variables. We determined household socioeconomic status using a previously validated scoring instrument that assessed material possessions, house structure, and access to food, water, and electricity [28].  We added this text to S2 Table  Model Diagnostics  While traditional measures of model fit (e.g., AIC or likelihood-ratio tests) are not directly available for GEE models due to their quasi-likelihood framework, we conducted diagnostic checks using a comparable Poisson GLM with robust standard errors to evaluate residual patterns and multicollinearity.  Diagnostic plots of deviance and Pearson residuals showed no evidence of strong outliers, skewed distributions, or model misspecification. Residuals were generally centered around zero, with no patterns suggesting poor model fit. Additionally, we assessed multicollinearity using variance inflation factors (VIFs). All adjusted VIFs were close to 1, indicating that collinearity among predictors was not a concern. These findings suggest that the model assumptions were reasonable and that our results are not likely biased by model misfit or predictor redundancy. | We thank the reviewer for this important comment.  We have included unadjusted and adjusted estimates in S2 Table. These estimates do not vary substantially, indicating that our results were not confounded by age or socioeconomic status.  We have explained our model diagnostics below S2 Table. |
| The results are presented solely in a dense table, which includes both prevalence rates and “ova concentrations” (actually gene copies per gram, as detected by PCR). However, this table format does not effectively convey key patterns or support interpretation. Visual summaries would significantly enhance clarity. |  | We agree that visual summaries can enhance clarity, but we have retained the table to provide more detail on our results to the reader. |
| Discussion |  |  |
| The manuscript frequently reiterates the use of “highly sensitive qPCR” without demonstrating how this influenced the study’s results or interpretation. The value of the molecular approach should be connected more explicitly to the study’s findings and public health implications, rather than being repeated as a methodological assertion. | We had deleted several uses of “highly sensitive” and included this sentence:  Line 235  qPCR has repeatedly been shown to be more sensitive than microscopy for diagnosing helminth infections, particularly in cases of low-intensity infections [36–38]. | Agreed |
| The authors briefly introduce other topics—such as enteric viruses and the gut microbiome—without linking them to their study objectives or results. This weakens the narrative and shifts attention away from the core findings. Similarly, the discussion of WHO morbidity targets is not well integrated. The authors acknowledge that qPCR cannot reliably define infection intensity, yet they proceed to suggest the data support progress toward those targets, which is contradictory. |  | While there are limitations to using qPCR to estimate infection intensity, there is a paucity of data on helminth infection and intensity in Uganda. While not the primary outcome of this study, we feel these data will also be of interest to readers of PLOS One and have choose to retain this information. |
| The conclusion does not provide a strong, evidence-based takeaway and does not clearly link back to the original aim of assessing whether intestinal helminth infections modify the risk of severe malaria. The interpretation should more directly address how the findings support or refute this objective. | We have added two sentences to the end of the discussion section:  Line 285  Our findings do not support a role for intestinal helminth infections in modifying the risk of severe malaria in young children in Kampala and Jinja. This suggests that in similar low-prevalence settings, intestinal helminths may be unlikely to contribute to the pathogenesis of severe malaria. |  |
