# Supplementary material for: Low prevalence of helminth infection in Ugandan children hospitalized with severe malaria
Source: PLoS One. 2025 Sep 11;20(9):e0332246. doi: 10.1371/journal.pone.0332246 (PMC12425322; doi:10.1371/journal.pone.0332246)
Supplement: Attachment — Submitted filename: Reviewer comments PONE-D-25-08603.pdf [file pone.0332246.s008.pdf]

Reviewer's comments
Manuscript title: Low prevalence of soil transmitted helminth infection in Ugandan children hospitalized with severe malaria

This manuscript addresses an important and timely question regarding the relationship between intestinal helminth infections and severe malaria among young children in Uganda. The study leverages a well-characterized cohort and employs modern molecular diagnostic techniques, which are strengths that contribute to the relevance and potential impact of the findings. The manuscript is clearly written in many places, and the main conclusion—that helminth infections were rare and not associated with severe malaria—is supported by the data.

However, several aspects of the manuscript require clarification and improvement to meet the standards of rigor and transparency expected for publication. Key concerns include insufficient detail in the methods section (particularly regarding cohort selection, control classification, and statistical modeling), lack of clarity in how outcomes were defined and analyzed, and inconsistencies between the stated aims and the interpretation of results. In addition, the discussion section lacks focus and does not fully engage with the study's limitations or contextual factors that may explain the observed findings. Addressing these issues will strengthen the manuscript's credibility and utility for readers.

Title

The title refers only to soil-transmitted helminths, but the study also includes *Schistosoma mansoni*, which is not classified as an STH. Consider revising the title to more accurately reflect the full range of intestinal helminths tested.

Abstract

The introduction provides a general background and identifies a gap in knowledge about the interaction between helminths and malaria severity. While the aim of the study is reasonably clear, several aspects of the abstract lack clarity, making it difficult to understand the study design, control group, and interpretation of the results. The rationale could be more clearly stated.

- It is unclear whether the community control children were tested for *Plasmodium* spp. infections. Were these controls truly malaria-negative, or simply asymptomatic?
- The use of children from the "same household or neighborhood" is reasonable, but this detail should be explained more thoroughly to ensure that the reader understands how confounding factors were minimized.
- The small number of controls (n=25 vs. 177 cases) also raises concerns about statistical power and should be mentioned or justified.

- The methods for helminth detection are well-described (stool sample testing using qPCR), but more clarity is needed on the malaria diagnosis—was it based on clinical presentation, lab confirmation, or both?
- The adjusted risk ratio (aRR = 1.0) and wide confidence interval suggest no effect, but the meaning of these statistics should be clearer for a broad audience. A plain-language interpretation would help, e.g., “Helminth infection did not increase or decrease the likelihood of severe malaria.”
- The concluding sentences introduce a key context (the deworming program in Uganda) which seems important but is not tied explicitly to the study’s findings until the end. Consider stating this earlier to help frame the findings.
- Also, "not a major contributor to risk" is vague—based on the data, it's more accurate to say helminths were not associated with severe malaria in this sample.

Introduction

The introduction provides extensive background on intestinal helminths and malaria, but it would benefit from more concise writing, better organization, and clearer articulation of the study’s rationale and framework. While the global and national context is appreciated, the current form sometimes overwhelms the reader with detail that could be streamlined. At the same time, key elements—such as the theoretical basis for helminth-malaria interactions, the relevance of the chosen cohort, and the specific methods—are underdeveloped.

- The rationale for the study—that helminths might modulate malaria risk—is acknowledged but underexplored. A few sentences explaining potential biological mechanisms (e.g., immune modulation by chronic helminth infection) would strengthen the logical bridge to the study aims.
- The line “determine if intestinal helminths modified risk of severe malaria” is somewhat vague. Clarify how modification of risk is measured—i.e., by comparing prevalence of helminths between cases and controls, and using adjusted risk ratios.
- The introduction of the “Neurodevelopment Outcomes in Children with Severe Malaria Study” (NDI) lacks context. Readers unfamiliar with the study may be confused by its sudden mention and unclear relevance to the helminth-malaria research question. A brief summary of what the NDI study is, and why it provides a suitable population for this analysis, would be helpful.

Methods

The methods section outlines an important study but lacks sufficient clarity and detail for reproducibility and rigorous interpretation. Critical information on participant selection,

sample processing, and statistical modeling is either vague or entirely missing. While the molecular procedures are clearly described, the epidemiological and analytic frameworks need revision.

- The description of cohort enrollment is convoluted and difficult to follow. Please clarify whether 600 children with severe malaria were enrolled or whether 600 children total were enrolled across both groups. The breakdown by group and recruitment site should be clearly tabulated.
- It is not clear whether community controls were tested for *Plasmodium* spp., or if they were assumed uninfected based on lack of symptoms. This distinction is crucial for interpreting any analysis of "risk modification."
- The phrase "nuclear family, extended family, or household area" is imprecise. Clarify whether community controls were biologically related, shared the household, or were simply neighborhood-matched.
- The selection of the 202 stool samples appears arbitrary, as the manuscript does not report how many total samples were available or how many were excluded based on timing or quantity. It is also unclear whether the final sample is representative of the broader cohort or if exclusions may have introduced bias.
- The molecular methods appear rigorous, but the repeated use of "highly sensitive" should be supported by data or comparison to alternative detection methods. What was the lower limit of detection for each helminth species?
- A table of primer targets or detection thresholds would enhance transparency (either in manuscript or supplementary materials).
- The power calculation is based on a 20% helminth prevalence drawn from outdated data (1998-2005) on school-aged children, which does not reflect the current study population of 6–48-month-olds or account for the impact of Uganda's national deworming program (started in 2003 expanded in 2005 through Child Health Days). Your own findings show a much lower prevalence, suggesting the study may be underpowered to detect the expected effect size. This weakens the basis for interpreting a null association. A post hoc power calculation using observed prevalence or a sensitivity analysis would help clarify the strength of your conclusions.
- The use of Poisson regression should be justified. If it was used to estimate relative risks for a binary outcome, did the authors apply a version of the model that adjusts for overdispersion or variance mis-specification? Given the binary nature of the outcome (severe malaria: yes/no), logistic regression may have been more appropriate and more conventional.

- Socioeconomic status is included as a covariate but not defined. How was it measured or categorized?
- Clustering by site is appropriate, but the modeling approach (e.g., fixed vs. random effects, multilevel modeling) is not explained.

Results

- The use of the term "ova concentration" in Table 1 is misleading given that the study employed PCR-based methods, which detect parasite DNA, not eggs. PCR quantifies nucleic acid regardless of whether it originates from eggs, larvae, or adult worms, and does not provide visual confirmation of ova as microscopy does. It would be more appropriate and scientifically accurate to report these results as "parasite DNA concentration" or "gene copies per gram of feces." This correction is important to avoid confusion about what was actually measured and how the findings should be interpreted.
- The use of t-tests to compare helminth prevalence and gene copy concentrations between sites (e.g., Kampala vs. Jinja) is not described in the methods and appears inconsistent with the stated statistical approach. Given that the authors accounted for clustering by site in their Poisson regression model, it is unclear why additional unadjusted site-level comparisons were performed.
- The Poisson regression model results are briefly presented, and the main finding—a non-significant association (aRR = 1.0, p = 0.78)—is clearly stated. However, the model output is summarized without context. For example, the confidence interval includes both potential protective and harmful associations, indicating high uncertainty. There is no discussion of model fit, multicollinearity, or residuals. In the supplementary table, multiple socioeconomic strata are included, but no explanation is given for how these were measured or categorized. Without that detail, it’s difficult to assess whether the covariate adjustment is meaningful or adequately controlled for confounding.
- The results are presented solely in a dense table, which includes both prevalence rates and “ova concentrations” (actually gene copies per gram, as detected by PCR). However, this table format does not effectively convey key patterns or support interpretation. Visual summaries would significantly enhance clarity.

Discussion

The discussion needs significant restructuring to improve focus, coherence, and scientific depth. While the authors correctly report a low prevalence of intestinal helminth infections,

the interpretation remains superficial and repetitive. There is limited effort to explore why helminth detection was so low.

- The manuscript frequently reiterates the use of "highly sensitive qPCR" without demonstrating how this influenced the study's results or interpretation. The value of the molecular approach should be connected more explicitly to the study's findings and public health implications, rather than being repeated as a methodological assertion.
- The authors briefly introduce other topics—such as enteric viruses and the gut microbiome—without linking them to their study objectives or results. This weakens the narrative and shifts attention away from the core findings. Similarly, the discussion of WHO morbidity targets is not well integrated. The authors acknowledge that qPCR cannot reliably define infection intensity, yet they proceed to suggest the data support progress toward those targets, which is contradictory.
- The conclusion does not provide a strong, evidence-based takeaway and does not clearly link back to the original aim of assessing whether intestinal helminth infections modify the risk of severe malaria. The interpretation should more directly address how the findings support or refute this objective.
